# New Rhenium-Doped SrCo_1−x_Re_x_O_3−*δ*_ Perovskites Performing as Cathodes in Solid Oxide Fuel Cells

**DOI:** 10.3390/ma9090717

**Published:** 2016-08-24

**Authors:** Loreto Troncoso, María Celeste Gardey, María Teresa Fernández-Díaz, José Antonio Alonso

**Affiliations:** 1 Institute of Materials Science of Madrid, CSIC, Cantoblanco, Madrid 28049, Spain; ja.alonso@icmm.csic.es; 2 Institute of Materials and Thermomechanics, Universidad Austral de Chile, General Lagos 2086, Valdivia 5111187, Chile; 3 Grupo CLIOPE, National Technological University, Reg. Mendoza, Rodríguez 273, Mendoza 5500, Argentina; mcgardey23@gmail.com; 4 Institut Laue-Langevin, B.P. 156x, Grenoble 38042, France; ferndiaz@ill.fr

**Keywords:** IT-SOFC, hydrogen, cathode, neutron diffraction, SrCoO_3_

## Abstract

In the aim to stabilize novel three-dimensional perovskite oxides based upon SrCoO_3−*δ*_, we have designed and prepared SrCo_1−x_Re_x_O_3−*δ*_ phases (x = 0.05 and 0.10), successfully avoiding the competitive hexagonal 2H polytypes. Their performance as cathode materials in intermediate-temperature solid oxide fuel cells (IT-SOFC) has been investigated. The characterization of these oxides included X-ray (XRD) and in situ temperature-dependent neutron powder diffraction (NPD) experiments for x = 0.10. At room temperature, SrCo_1−x_Re_x_O_3−*δ*_ perovskites are defined in the P4/mmm space group, which corresponds to a subtle tetragonal perovskite superstructure with unit-cell parameters a = b ≈ a_o_, c = 2a_o_ (a_o_ = 3.861 and 3.868 Å, for x = 0.05 and 0.10, respectively). The crystal structure evolves above 380 °C to a simple cubic perovskite unit cell, as observed from in-situ NPD data. The electrical conductivity gave maximum values of 43.5 S·cm^−1^ and 51.6 S·cm^−1^ for x = 0.05 and x = 0.10, respectively, at 850 °C. The area specific resistance (ASR) polarization resistance determined in symmetrical cells is as low as 0.087 Ω·cm^2^ and 0.065 Ω·cm^2^ for x = 0.05 and x = 0.10, respectively, at 850 °C. In single test cells these materials generated a maximum power of around 0.6 W/cm^2^ at 850 °C with pure H_2_ as a fuel, in an electrolyte-supported configuration with La_0.8_Sr_0.2_Ga_0.83_Mg_0.17_O_3−_*_δ_* (LSGM) as the electrolyte. Therefore, we propose the SrCo_1−x_Re_x_O_3−*δ*_ (x = 0.10 and 0.05) perovskite oxides as promising candidates for cathodes in IT-SOFC.

## 1. Introduction

Many efforts are being devoted to design and prepare novel cathode materials for solid oxide fuel cells (SOFC) able to perform at intermediate temperatures (550–850 °C) [1,2,3,4,5]. A decrease of the operation temperature means a reduction of the electrode kinetics and an increase of the interfacial polarization resistances. The oxygen-reduction reaction (ORR) is, in particular, severely affected at the cathode material [5]. A frequently used strategy to minimize the polarization resistance at lower temperatures between the cathode and electrolyte interface, and to improve the behavior of the electrodes, is to develop “mixed ionic and electronic conductors” (MIEC [6]) able to perform at the whole superficial area and not only at the triple-phase boundaries (TPB).

A promising cathode material is the high-temperature 3C cubic phase of SrCoO_3−*δ*_ perovskite, where 3C stands for a cubic stacking of AO_3_ units yielding a corner-sharing arrangement of BO_6_ octahedra in the nominal ABO_3_ perovskite structure due to its high electrical conductivity. This material is able to host a considerable number of oxygen vacancies, allowing a notable oxygen permeation flux [7,8]. Unfortunately, this does not occur in the competitive 2H hexagonal polytype phase, which is easily stabilized below 900 °C when cooling in air the 3C cubic perovskite. This hexagonal phase has an extremely poor transport behavior due to the columns of octahedra CoO_6_ sharing faces along the c-axis. The stabilization of a 3C perovskite framework in the SrCoO_3−*δ*_ system at room temperature has been achieved through several chemical substitutions that have been performed either at the Sr (Sr_x_M_1−x_CoO_3−*δ*_) or at the Co positions (SrCo_x_M_1−x_O_3−*δ*_), with atoms such as Ba, La, Sm and Sc, Fe, Ni, and Mn, respectively [9,10,11,12]. A particularly successful strategy is the introduction of a small fraction of highly charged cations at the Co sublattice in order to destabilize the columns of CoO_6_ octahedra sharing faces that constitute the competitive 2H polytype. Following previous investigations on SrCo_1−x_M_x_O_3−*δ*_ (M = Sb, Mo, Nb, V, Ti) [13,14,15,16], in this work we have successfully explored the introduction of M = Re^6+^ at the Co site, since Re is well known to occupy the octahedral positions in many perovskite-related compounds [17,18,19].

The aim of this paper is to correlate the performance observed for Re-doped materials as cathodes in SOFC with the structural features, determined in a comprehensive neutron powder diffraction (NPD) structural study. We report on the preparation and characterization including the electrical conductivity, polarization resistance in symmetrical cells, dilatometry, and performance in single test cells working with pure hydrogen as fuel.

## 2. Experimental

SrCo_1−x_Re_x_O_3−*δ*_ (x = 0.00, 0.05, and 0.10) were synthesized via a nitrate–citrate route. The starting materials Sr(NO_3_)_2_, Co(NO_3_)_3_·6H_2_O, and ReO_3_ of analytical grade were dissolved under stirring in 250 mL of 10% citric acid aqueous with 20 mL of concentrated HNO_3_. The pH of the final solution was 1. The resin was dried at 120 °C and slowly decomposed at temperatures up to 600 °C for 12 h. A final treatment in air at 1100 °C for 12 h for each compound was carried out to ensure the complete crystallization of the samples.

The reaction products were characterized by X-ray diffraction (XRD) for phase identification and for assessment of the phase purity using a Brucker D8 diffractometer (Madrid, Spain) (40 kV, 30 mA), controlled by a DIFFRACT^PLUS^ software (Bruker Española S.A., Madrid, Spain), in Bragg-Brentano reflection geometry with Cu K*α* radiation (*λ* = 1.5418 Å), between 10 and 90 angular range. Neutron powder diffraction (NPD) data were collected for SrCo_0.9_Re_0.1_O_3−*δ*_, using the D2B diffractometer at Institut Laue-Langevin, Grenoble, in the high-resolution configuration with a neutron wavelength *λ* = 1.549 Å. About 2 g of the sample was contained in a vanadium can for the room temperature (RT) data collection, and in a quartz tube open to the air for the temperature-dependent experiments, trying to mimic the actual working conditions of a cathode material in air. This sample holder was placed in the isothermal zone of a furnace with a vanadium resistor operating under vacuum (P_O2_ = 10^−6^ Torr). The measurements were carried out at 25, 400, 600, 800 and 950 °C. The counting time for each pattern was 2 h. Diffraction data were analyzed by the Rietveld method [20], using the FULLPROF refinement program [21]. A pseudo-Voigt function was considered to generate the profile shape. The following parameters were refined in the final run of the fit: scale factor, background coefficients, zero-point error, unit-cell parameters, pseudo-Voigt corrected for asymmetry parameters, positional coordinates, isotropic thermal factors for the metals and anisotropic for O1, O2 and O3 oxygen atoms. Occupancy factors for oxygen atoms were also refined from NPD data. The coherent scattering lengths of Sr, Co, Re and O were 7.02, 2.49, 9.20 and 5.803 fm, respectively.

Thermal analysis were realized in a ATD/DSC/TG model Q600 TA Instruments, from 25 °C to 900 °C with a ramp of 10 °/min.

Thermal expansion coefficient and *dc* electrical conductivity measurements were carried out for SrCo_1−x_Re_x_O_3−*δ*_ (x = 0.05, 0.10) samples in sintered samples. Densifications were performed by uniaxial pressing at 200 MPa for 5 min to form green pellets that were subsequently calcined at 1100 °C. Thermal expansion was performed in a dilatometer Linseis L75HX1000 (Linseis Thermal Analysis, Madrid, Spain), between 25 and 850 °C in air atmosphere in disk-shaped pellets. The *dc* conductivity was measured between 25 and 850 °C in air by the four-point method in bar-shaped pellets under *dc* currents between 0.01 and 0.3 A. *AC* electrical conductivity was measured for SrCo_1−x_Re_x_O_3−*δ*_ (x = 0.00, 0.05 and 0.10) by impedance spectroscopy (IS). IS measurements were performed in air under open-circuit potential (OCP) conditions in a symmetrical configuration to extract the corresponding values of the electrolyte and electrode contributions. For this purpose, a dense electrolyte pellet of La_0.9_Sr_0.1_Ga_0.83_Mg_0.17_O_3−*δ*_ (LSGM was used). LSGM was prepared in our laboratory by a solid state synthesis. The starting materials La_2_O_3_, MgO, SrCO_3_ and Ga_2_O_3_ were mixed, pressing into pellets and heated at 1000 °C for 20 h, then at 1200 °C for 20 h and finally sintered at 1450 °C for 20 h.

In this case, dense compounds are required between 90% and 95%. The densities of our electrolytes were 95%. The densities were determined using Equations (1) and (2):

*ρ* = m/V (1)

*ρ*_x_ = Z·W/V·Nav (2)

where *ρ* and *ρ*_x_ are the experimental and theoretical density of the sample, respectively. The percentage of the degree of densification is obtained as (*ρ*/*ρ*_x_) × 100.

The powder SrCo_1−x_Re_x_O_3−*δ*_ (x = 0.00, 0.05 and 0.10) materials were ball-milled in ethanol to break the agglomerates. The dried powders were then mixed with a binder (V-006 from Heraeus, Madrid, Spain) to create an ink of the cathode material. The ink was symmetrically painted over the electrolyte onto both surfaces in configuration SrCo_1−x_Re_x_O_3−*δ*_/LSGM/SrCo_1−x_Re_x_O_3−*δ*_ (x = 0.00, 0.05 and 0.10). The cells were calcined at 1100 °C for 4 h to obtain a good adherence between the cathodes and the electrolytes according to a procedure previously optimized in our laboratories for doped-SrCoO_3_ cathode powders. Subsequently, two Pt electrodes were painted onto the cathode surfaces and calcined at 800 °C for 1 h to ensure equipotential conditions. IS was then performed in potentiostatic mode, decreasing the temperature from 850 to 750 °C, with an excitation voltage of 50 mV in the range of 1 kHz to 100 mHz. All the cell impedances were normalized by the superficial area so that they have the units of Ω·cm^2^. The data obtained were analyzed with the Zview software (version 3.4c, Scribner Associates, Inc., Hampshire, UK) [22]. The *ac* and *dc* currents were applied and collected with a Potentiostat-Galvanostat AUTOLAB (PGSTAT 302 and FRA2) module from ECO CHEMIE.

Fuel single-cell tests were made on electrolyte-supported configuration with LSGM as the electrolyte, SrCo_1−x_Re_x_O_3−*δ*_ (x = 0.05, 0.10) (SCRO) as cathode materials and SrMo_0.8_Fe_0.2_O_3−*δ*_ (SMFO) as anode material, recently developed in our group [23]. This anode present a metallic type conductivity behavior with values above 120 S·cm^−1^ to normal operating temperatures and a maximum power density of 790 m·W/cm^2^ at 850 °C with pure H_2_ as fuel. SMFO was prepared by dissolving stoichiometric amounts of Sr(NO_3_)_2_, (NH_4_)_6_Mo_7_O_24_∙2H_2_O and C_2_FeO_4_∙4H_2_0 and citric acid (10% solution). Upon decomposition of the resins to 600 °C for 12 h in air, scheelite structure was identified by X-ray diffraction with Mo (VI). A final treatment at 1050 °C in a tube furnace with a flow of H_2_/N_2_ (5%/95%) for 15 h led to the formation of perovskite oxides required. La_0.4_Ce_0.6_O_2−*δ*_ (LDC) was used as a buffer layer between the anode and the electrolyte in order to prevent the interdiffusion of ionic species. Inks of LDC, SMFO and SCRO were prepared with a binder (V-006 from Heraeus). LDC ink was screen-printed onto one side of the LSGM disk followed by a thermal treatment at 1300 °C in air for 1 h, which it is the time necessary to achieve the coupling of the materials. SMFO was subsequently screen printed onto the LDC layer and fired at 1100 °C in air for 1 h. SCRO was finally screen printed onto the other side of the disk and fired at 1100 °C in air for 1 h. The working electrode area of the cell was 0.25 cm^2^ (0.5 × 0.5 cm). Pt gauze with a small amount of Pt paste was used as current collector at both the anodic and the cathodic sides for ensuring electrical contact. The cells were tested in a vertical tubular furnace at 800 and 850 °C; the anode side was fed with pure dry H_2_, whereas the cathode worked in an air flow. The fuel-cell tests were performed with an AUTOLAB 302N Potentiostat/Galvanostat by changing the voltage of the cell from the OCV (Open circuit voltage) to 0.1 V, with steps of 0.010 V, holding 10 s at each step. The current density was calculated by the recorded current flux through the effective area of the cell (0.25 cm^2^).

## 3. Results and Discussion

### 3.1. Crystallographic Characterization

The oxides of stoichiometry SrCo_1−x_Re_x_O_3−*δ*_ (x = 0.00, 0.05 and 0.10) were obtained as black, well-crystallized powders. No impurity phases were detected. A crystallite size analysis was made using the Williamson-Hall technique, finding a crystallite size of 34.5 nm for x = 0.05 and x = 0.10, while for x = 0.0 the size was 43.47 nm.

**Figure 1:**
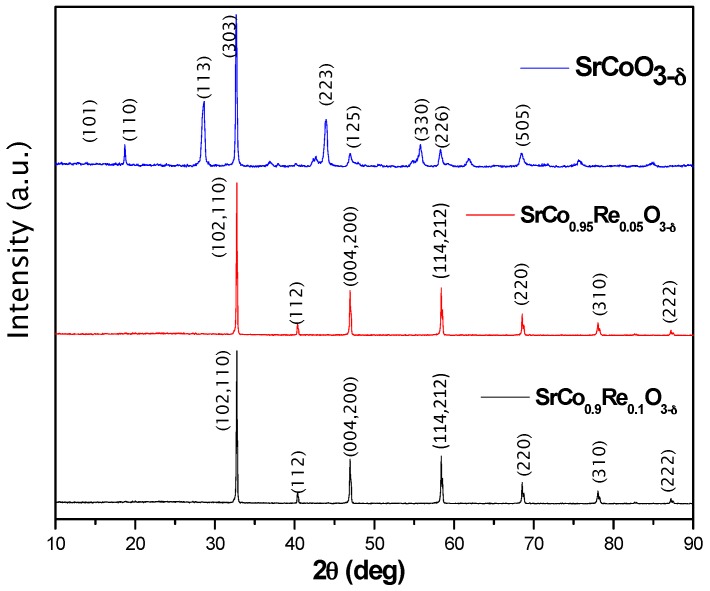
X-ray diffraction (XRD) patterns (Cu K*α*) for SrCo_1−x_Re_x_O_3−*δ*_. Pristine SrCoO_3−*δ*_ is indexed in the 2H hexagonal phase, while x = 0.05 and 0.10 oxides may be indexed in cubic Pm-3m symmetry.

**Figure 2:**
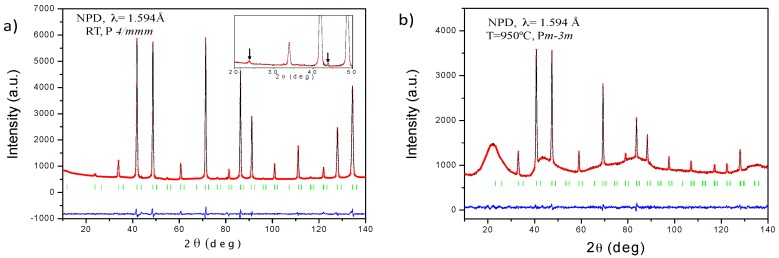
Observed (red crosses), calculated (black line) and difference (bottom line) NPD Rietveld profiles at RT for SrCo_0.90_Re_0.10_O_3−*δ*_ at: (**a**) RT refined in the P4/mmm space group, and; (**b**) 950 °C refined in the cubic Pm-3m.

The laboratory XRD diagrams at room temperature (RT) of the three compounds are shown in Figure 1. SrCoO_3−*δ*_ shows the unwanted 2H hexagonal phase, while SrCo_0.95_Re_0.05_O_3−*δ*_ and SrCo_0.9_Re_0.1_O_3−*δ*_ displays a characteristic diagram of a perovskite-type structure that can be indexed as a cubic unit cell with a_o_ = 3.86 Å and a_o_ = 3.87 Å for x = 0.05 and 0.10, respectively. A neutron study was essential to unveil the actual symmetry and space group of these perovskites. A NPD diagram was collected at RT for the SrCo_0.9_Re_0.1_O_3−*δ*_ compound as shown in Figure 2a. The presence of tiny superstructure peaks (inset of Figure 2a) required to consider a tetragonal unit cell with doubled c parameter, as a = b ≈ a_o_, c = 2a_o_. All the reflections can be indexed in the P*4/mmm* space group (No. 123), and the crystal structure was refined in a model derived for SrCo_0.9_Sb_0.1_O_3−*δ*_ [24].

In this model, Sr atoms are located at 2*h* (1/2,1/2,z) sites, Co and Re atoms are distributed at random at 1*a* (0,0,0) and 1*b* (0,0,1/2) positions and the oxygen atoms O1 at 2*e* (1/2,0,0), O2 at 2*g* (0,0,z) and O3 at 2*f* (1/2,0,1/2) sites. After refinement of the mixed occupancy factors of Co and Re at 1*a* and 1*b* sites, it was evident that Re atoms all occupy 1*b* sites. The occupancy factors for the three types of oxygen atoms were also refined. For O2, the occupancy factors show full occupancy. However, for O1 and O3 we find a significant oxygen deficiency, as indicated in Table 1, corresponding to a total *δ* = 0.155(2), i.e., at RT we obtained a crystallographic formula Sr(Co_0.9_Re_0.1_)O_2.845(2)_ from the NPD refinement. Figure 2a illustrates the goodness of the fit for the RT pattern. Table 1 lists the final structural parameters and discrepancy factors. Thethe main interatomic distances for RT from NPD data are Co1–O1(x4): 1.93382(5), Co1–O2(x2): 1.898(12), (Co,Re)2–O2(x4): 1.966(12) and (Co,Re)2–O3(x2): 1.93382(5). The equatorial distances Co1-O1 and (Co,Re)2–O3 exhibit the same value of 1.9338(1) Å, whereas the axial Co1-O2 bond lengths are significantly shorter, of 1.898(12) Å, in flattened octahedra, and the axial (Co,Re)2–O2 bonding distance is considerably longer, 1.966(12) Å, conforming elongated (Co,Re)O_6_ octahedra.

Therefore, there is an alternation of small (Co1O_6_) and large (Co_2_O_6_) octahedra, where the short-long-short arrangement of the Co-O bond lengths suggests the establishment of a charge disproportionation or charge ordering effect across both types of Co cations. These differences in the octahedral size suggest that Co_1_ cations may present a higher oxidation state than Co_2_. Assuming that Re adopts an oxidation state of 6+, stable in the synthesis conditions in air, in Sr(Co_0.9_Re_0.1_)O_2.845(2)_ perovskite Co ions show an average valence of 3.43+ at RT, thus the assumption of a full charge disproportionation would mean an average oxidation state of Co^3.73+^ at Co1 sites and Co^3+^ at Co2 positions. Therefore, the occurrence of intermediate spin (IS) Co^3+^ at Co2 sites would account for the Jahn-Teller elongation of the Co-O bond-lengths, as observed, inducing a shift of O^2−^ towards the Co^4+^ cations, thus from Co_2_ to Co_1_.

It was interesting to study the structural evolution of the Sr(Co_0.9_Re_0.1_)O_3−*δ*_ at the working conditions of a SOFC, which was investigated in an in-situ temperature-dependent NPD experiment at 400, 600, 800 and 950 °C. At 400 °C and above, the NPD data could be indexed in a simple-cubic perovskite unit cell. This was evident from the disappearance of the superstructure reflections arising from the doubling of the unit cell along the c-direction; the high-temperature crystal structures were refined in the Pm-3m space group, where Sr atoms were located at 1*b* (1/2,1/2,1/2) positions, Co and Re distributed at random over the 1*a* (0,0,0) sites and a single type of oxygen atom at 3*d* (1/2,0,0). The occupancy factor for the oxygen was also refined, finding a higher deficiency as temperature increased. The *δ* values are *δ* = 0.267(2), *δ* = 0.292(3), *δ* = 0.351(3) and *δ* = 0.434(3) at 400, 600, 800 and 950 °C, respectively. Figure 2b illustrates the goodness of the fit for the 950 °C pattern. The irregular background comes from the quartz container utilized to maintain the cathode material in contact with the air atmosphere. Table 2 lists the final structural parameters, discrepancy factors and the main interatomic distances obtained from 400 to 950 °C NPD data. As expected, the (Co,Re)–O distances become longer as temperature increases.

**Figure 3:**
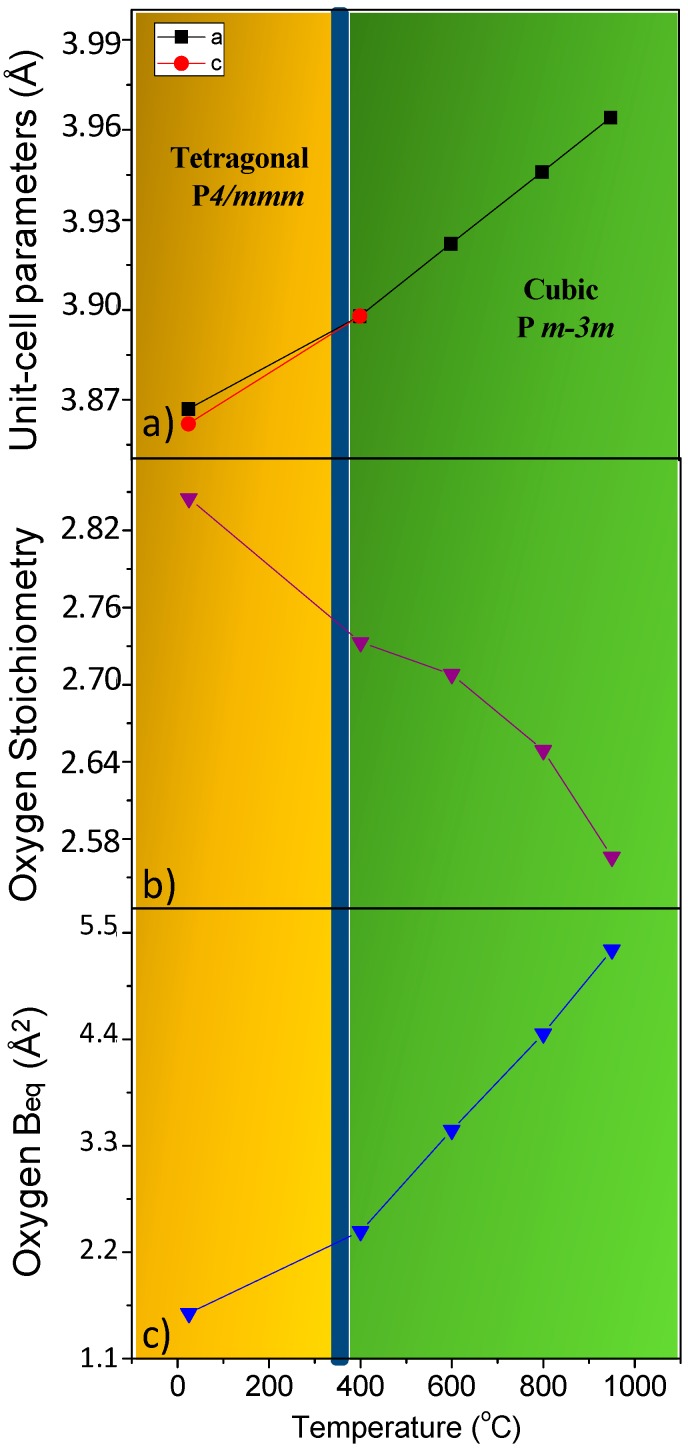
(**a**) Thermal variation of the unit cell parameters of SrCo_0.90_Re_0.10_O_3−*δ*_ from in situ NPD data. The phase transition from tetragonal to cubic occurs at 385 °C as determined from dilatometric measurements; (**b**) Oxygen stoichiometry of SrCo_0.90_Re_0.10_O_3−*δ*_ from in situ NPD data; (**c**) Thermal variation of the equivalent thermal displacement factor (B_eq_) for oxygen atoms.

Figure 3a displays the thermal variation of the unit cell parameters, showing the transition from a low-temperature tetragonal superstructure to the high-temperature simple-cubic symmetry. As it will be shown below, the dilatometric measurements indicate that the transition temperature takes place at 385 °C. This phenomenology was also presented by the SrCo_0.9_Sb_0.1_O_3−*δ*_ perovskite, which exhibits a tetragonal-to-cubic transition at a considerably higher temperature range (700–850 °C) [25].

Figure 3b shows the variation of the oxygen stoichiometry of the perovskite, which appreciably decreases with temperature, thus providing a large amount of oxygen vacancies at the working conditions of a SOFC, enabling the diffusion of oxide anions across the solid. On the other hand, Figure 3c presents the thermal variation of the equivalent thermal displacement factor (B_eq_) for oxygen atoms, which noticeably increases with temperature, accounting for the large mobility expected for oxide ions at high temperatures; it is noteworthy that the slope increases at the phase transition, implying a better lability in the cubic phase.

**Figure 4:**
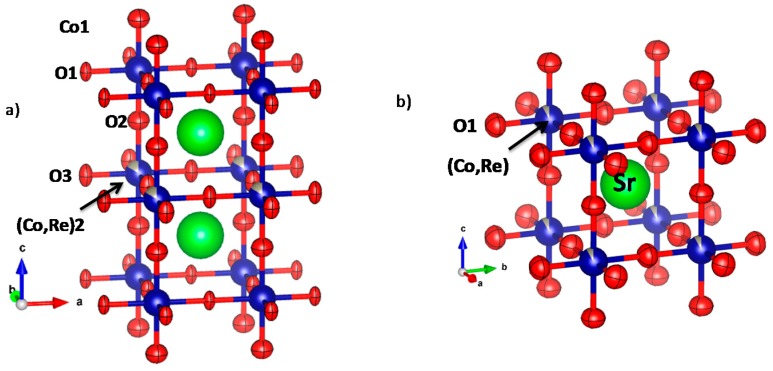
(**a**) Tetragonal crystal structure observed for SrCo_0.90_Re_0.10_O_3−*δ*_, where an array of corner-linked Co_1_O_6_ octahedra alternates along the c axis with layers of (Co,Re)2O_6_ octahedra; (**b**) High-temperature structure corresponding to the cubic aristotype.

In all cases (low-temperature tetragonal and high-temperature cubic structures) an anisotropic refinement of the thermal displacement factors was introduced to describe the thermal motions of the oxygen atoms. Figure 4a,b shows a view of the crystal structures highlighting the orientation of the ellipsoids for the tetragonal (at RT) and cubic (at 950 °C) phases. For the tetragonal structure, the thermal displacements for equatorial O1 and O3 atoms are quite anisotropic, with cigar-shaped ellipsoids perpendicular to the Co-O-Co direction. This may suggest a dynamical tilting of the Co_2_O_6_ octahedra, which may be a hint of the migration of the oxygen vacancies along the c axis, shortening and stretching the O-O distances along this direction. Axial O_2_ oxygen atoms are found to be much less anisotropic, the long axes of the ellipsoids also being perpendicular to the bonding direction. The same arrangement is observed for the cubic structures (Figure 4b), where the thermal motion of O_1_ is perpendicular to the Co-O bonds.

### 3.2. Thermal Analysis, Thermal Expansion Measurements and Chemical Compatibility

**Figure 5:**
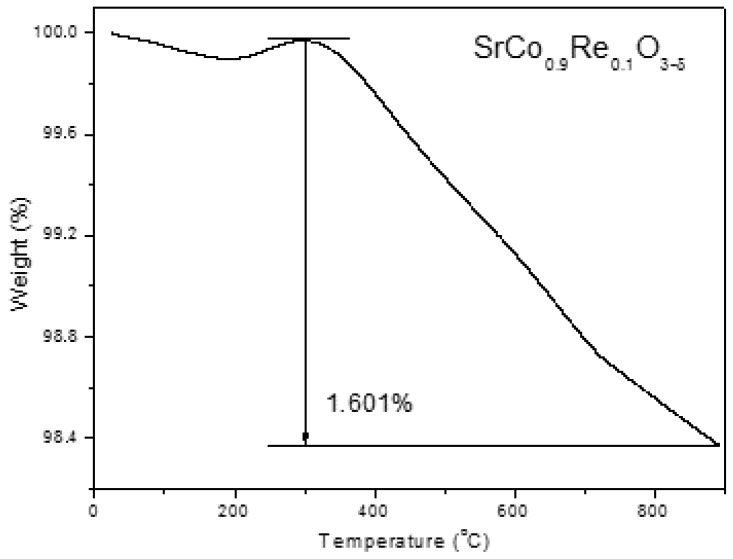
Thermal analysis in air (Thermogravimetric curve) for SrCo_0.9_Re_0.1_O_3−*δ*_.

The thermal evolution of the doped samples was studied by thermogravimetry analysis in air. Figure 5 shows the results for the x = 0.1 compound. At around 300 °C a weight gain is observed that could be related to the phase transition from tetragonal to cubic. Above this temperature, the perovskite suffers a continuous reduction without abrupt changes in weight loss. At 850 °C the weight loss is 1.54%. This means that the oxygen content at this temperature is 2.648, which is practically the same value obtained by NPD. The total weight loss is 1.601% and is reversible when cooling down.

**Figure 6:**
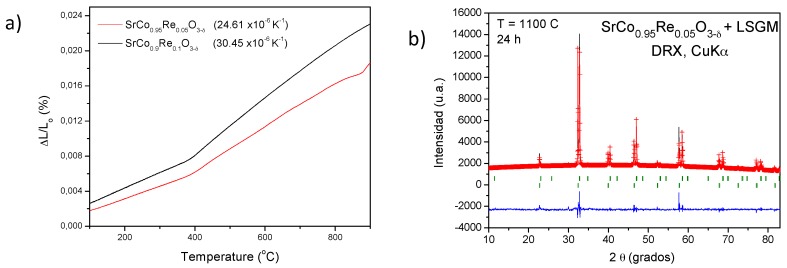
(**a**) Thermal expansion determined by dilatometry for SrCo_1−x_Re_x_O_3−*δ*_ (x = 0.05 and 0.10) perovskite; (**b**) XRD pattern after a treatment of SrCo_0.95_Re_0.05_O_3−*δ*_ and LSGM at 1100 °C for 12 h, showing no reaction products.

The Thermal Expansion Coefficient (TEC) is useful to determine the mechanical compatibility of our materials with the other cell components. A dilatometric analysis was performed between 25 and 850 °C for several cycles for a cylindrical sintered pellets (5 mm diameter × 2 mm thickness); the data where only recorded during the heating runs. Figure 6 shows the thermal expansion of SrCo_1−x_Re_x_O_3−*δ*_ (x = 0.05 and 0.10). From the slope of the L vs. T variation in the range of existence of the cubic-phase (400–950 °C) the thermal expansion coefficient is determined as

TEC = [(L_TF_ − L_Ti_)/L_Ti_]/(T_F_ − T_i_) (3)

where L represent the length; TF is the final temperature and Ti is the initial temperature.

The dilatometric analysis shows a variation in the slope for both compounds at 385 °C, which could be related to the change in the symmetry of the structure observed from NPD data for x = 0.1 perovskite. The TEC values are 24.61 × 10^−6^ K^−1^ and 30.45 × 10^−6^ K^−1^ for x = 0.05 and x = 0.10, respectively; the last figure is in excellent agreement with that determined for x = 0.1 oxide in the cubic phase domain from NPD data. In any case, those values are substantially higher than those usually displayed by SOFC electrolytes (LSGM, TEC = 12.5 × 10^−6^ K^−1^) [26] and it is typical of Co-based perovskites [27]. This high value could be minimized in composites with the electrolyte.

The chemical compatibility of SrCo_1−x_Re_x_O_3−*δ*_ (x = 0.05 and 0.10) with the LSGM electrolyte has also been checked by firing mixtures of both powdered materials at 1100 °C in air atmosphere for 12 h. Figure 5b shows a XRD analysis of the product, consisting of a mixture of unaltered perovskite phases of SrCo_0.95_Re_0.05_O_3−*δ*_ and LSGM, which is essential for the good performance of the cathode material during the cell operation.

### 3.3. Electrical Conductivity Measurements

**Figure 7:**
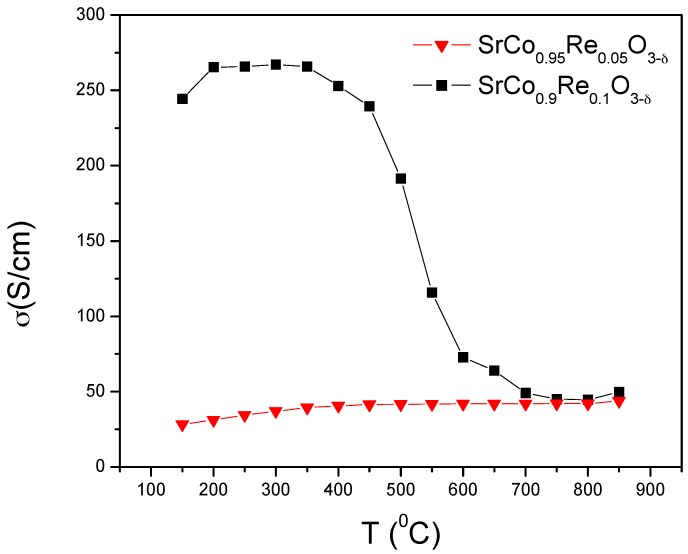
*DC* conductivity as a function of temperature for SrCo_1−x_Re_x_O_3−*δ*_ (x = 0.05 and 0.10).

Figure 7 shows the thermal variation of the electrical conductivity of SrCo_1−x_Re_x_O_3−*δ*_ (x = 0.05 and 0.10) measured in sintered bars (10 mm large × 3 mm width × 3 mm high) in air atmosphere by the DC four-probe method in the temperature range 100–850 °C; the bars were sintered during 12 h at 1000 °C. The compound SrCo_0.9_Re_0.1_O_3−*δ*_ shows a conductivity as high as 270 S·cm^−1^ at 400 °C, in the domain of existence of the tetragonal phase, and then a metal-insulator transition at 450 °C; the conductivity at 850 °C is above 52 S·cm^−1^. The perovskite SrCo_0.95_Re_0.05_O_3−*δ*_ instead shows a semiconducting-like conductivity; which rises up to 44 S cm^−1^ at 850 °C. In both cases, the conductivity at the working temperature of a SOFC is similar to that of the most widely used derivatives of SrCoO_3−*δ*_; for instance, Ba_0.5_Sr_0.5_Co_0.8_Fe_0.2_O_3−*δ*_ (BSCF) has a conductivity of 35 S·cm^−1^ at 850 °C [28], whereas SrCo_0.8_Fe_0.2_O_3−*δ*_ (SCFO) displays a *σ* of ~48 S·cm^−1^ at 850 °C [29].

### 3.4. Impedance Spectroscopy Results

**Figure 8:**
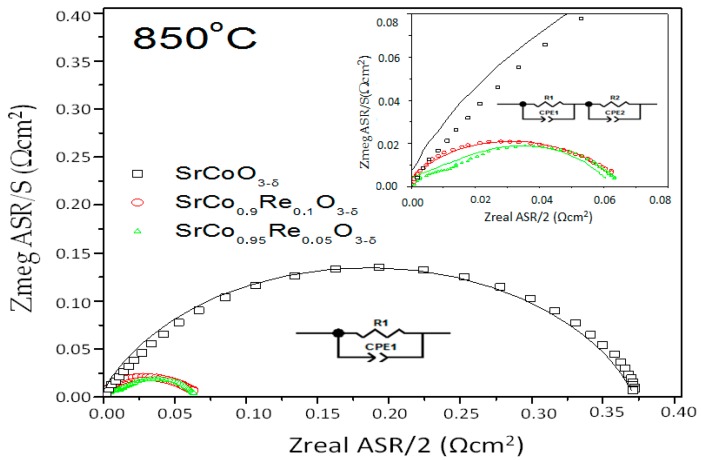
Impedance spectra obtained at 850 °C in symmetrical cells of SrCo_1−x_Re_x_O_3−*δ*_ (x = 0.00, 0.05 and 0.10) over La_0.8_Sr_0.2_Ga_0.83_Mg_0.17_O_3−*δ*_ electrolyte.

The electrode performance was first evaluated by impedance spectroscopy in air under OCP conditions with the same electrode on opposite sides of the LSGM electrolyte. Figure 8 shows the impedance spectra obtained at 850 °C for SrCo_1−x_Re_x_O_3−*δ*_ (x = 0.0, 0.05, 0.10).

Generally, the charge conduction processes in these materials are characterized by a resistive type process (impediment to charge circulation), and by a capacitive type process (“double layer” effect), both acting in parallel. Thus, each of the transport processes usually are simulated as an (RC) circuit. This two contributions correspond to a semicircumference and they are represented in a Nyquist diagram. The different charge conduction processes act as a sum of series process. This means that in an equivalent circuit this can be expressed as successive elements in (RC) series. Our experimental data resembles quite to this kind of circuit, but the center of the semicircle it is not found in the x-axis. Therefore, instead of using an ideal capacitor, a constant phase element (CPE) is used. CPEs represent a time-dependent capacitive element (pseudo-capacitance).

All the spectra measured include an ohmic resistance (intercept between the impedance arc at high frequencies and the real axis) mainly originating from the electrolyte, and the polarization resistance that can be determined as the sum of the resistances of each individual process, which corresponds to depressed arcs in the impedance spectrum (difference between high frequency and low frequency intercept with the real axis).

For x = 0.05 two arcs are visible, as it can be observe in the inset of Figure 8. Here the spectra was simulated by a (R1, CPE1-R2, CP2) circuit. This means that two processes are related to the mechanism of oxygen incorporation in the material (oxygen reduction reaction and transfer of ions at the interface). This same model was used to calculate the area specific resistance (ASR) at 700 °C of the 10 mol % Re-containing sample. At higher temperatures, only one arc can be observed for this compound (x = 0.1). In this case, the spectra were simulated by (R1, CPE1) elements. Finally, for x = 0.0 was observed only one arc in the entire temperature range. The results show good agreement between the experimental and fitted data.

Is possible to say that the sample of x = 0.05 presents a most resistive oxygen reduction reaction. The term used to describe the resistances associated with the electrolyte was subtracted from the spectra in order to have an estimation of the electrode polarization as a function of temperature. The polarization resistance in the SrCo_1−x_Re_x_O_3−*δ*_ electrolyte interface, referred to as Rp = R1 + R2, is the area specific resistance, ASR. All impedance diagrams were normalized by the superficial area, so the *R* parameters obtained in the fitting for each process were divided by two. The resistances *ASR* associated with the kinetics of electrode processes decrease with increasing temperature, as expected.

Table 3 summarizes the fitting parameters as a function of temperature for SrCo_1−x_Re_x_O_3−*δ*_/LSGM/SrCo_1−x_Re_x_O_3−*δ*_ cell at different temperatures in air under zero DC conditions, as well as the values of area specific resistance.

The good performance of SrCo_1−x_Re_x_O_3−*δ*_ as a cathode with the LSGM electrolyte is evidenced by the low electrode polarization resistances Those values are similar, especially at 850 °C, for the same class of perovskites doped with Ti or V used as cathodes described in previous works. The Ti-doped sample presents a 0.016 Ω·cm^2^ and the V-doped a 0.025 Ω·cm^2^ [30]. As can be seen, our results are slightly larger. This may be due to the fact that the Re^6+^ is more massive and has a larger ionic radius compared to Ti^4+^ and V^5+^ cations, generating a greater distortion in the perovskite B site, affecting the polarization resistence of the cathodes. It is interesting to evaluate the performance of pristine SrCoO_3−*δ*_ (2H hexagonal phase), also included in Figure 8 for the sake of comparison, at 850 °C. The electrode polarization in this case is: 4.522, 0.659 and 0.362 Ω·cm^2^ for temperatures of 750 °C, 800 °C, and 850 °C, respectively. These values are much higher (by an order of magnitude at 850 °C) than those obtained in the cubic phase, which demonstrates that the performance of pristine SrCoO_3−*δ*_ as a cathode would be much poorer. Such a high resistance indicates a difficulty for the oxygen ions to get to the electrolyte, in detriment to the electrochemical reaction (oxygen reduction) that is expected to catalyze a performing cathode material.

**Figure 9:**
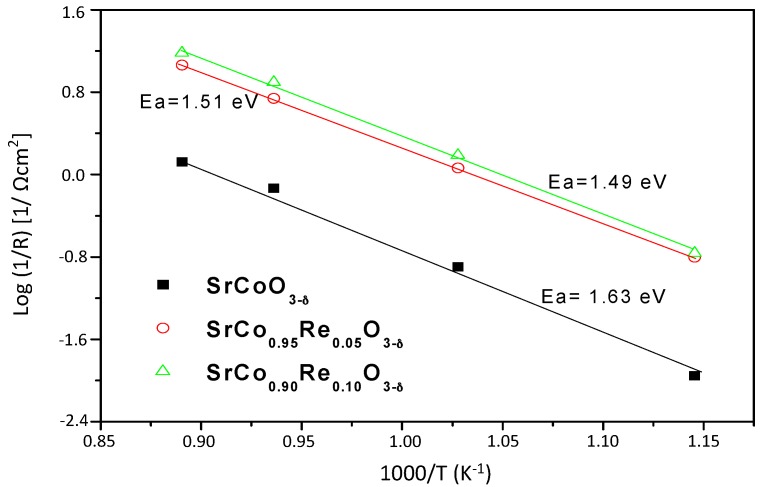
Arrhenius plots for the SrCo_1−x_Re_x_O_3−*δ*_/LSGM interface.

The activation energy can be obtained from the Arrhenius plots of the resistance as a function of temperature, as can be seen in Figure 9. The activation energies for x = 0.05 and x = 0.10 arcs are 1.51 and 1.49 eV, respectively. These values of activation energy are associated with the processes associated with oxygen reduction reaction.

### 3.5. Fuel-Cell Tests

**Figure 10:**
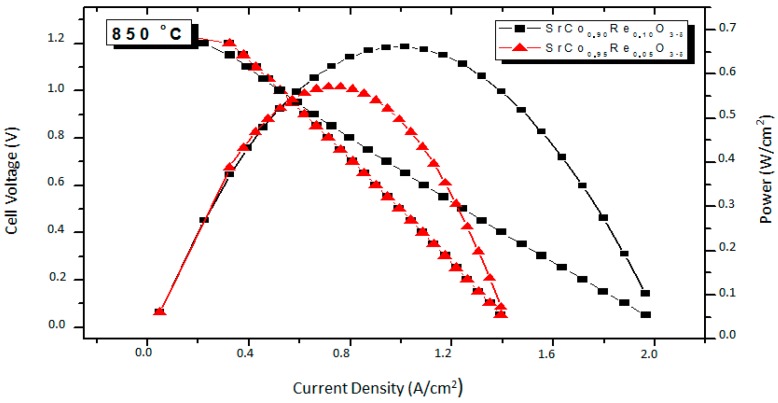
Cell voltage (left axis) and power density (right axis) as a function of the current density for the test cell with the configuration SrMo_0.8_Fe_0.2_O_3−*δ*_*/*La_0.4_Ce_0.6_O_2−*δ*_*/*La_0.9_Sr_0.1_Ga_0.83_Mg_0.17_O_3−*δ*_*/*SrCo_1−x_Re_x_O_3−*δ*_ in pure H_2_ measured at 850 °C with Pt as current collector.

The performance of the SrCo_1−x_Re_x_O_3−*δ*_ (x = 0.10 and 0.05) oxides as a cathode materials was tested in single cells in an electrolyte-supported configuration using a 300-μm-thick LSGM electrolyte. Figure 10 illustrates the cell voltage and power density as a function of current density at 850 °C for the single cells fed with pure H_2_. The cathode side is in direct contact with air atmosphere. The maximum power densities generated by the cell are 0.57 and 0.66 m·W·cm^−2^, for x = 0.05 and 0.10, respectively. This test confirms the good performance of these cathode materials, comparable to those reported for similar systems like SrCo_1−x_Sb_x_O_3−*δ*_ [23]. The stabilization of a three-dimensional network of corner-sharing octahedra superstructure with a 3C perovskite structure (cubic at the working conductions of the test cell) is achieved by replacing 5% or 10% Co by Re in the parent SrCoO_3−*δ*_ perovskite, thus avoiding the stabilization of the unwanted 2H hexagonal phase. The observed performance as a mixed ionic-electronic conductivity (MIEC) oxide, combining excellent ionic and electronic conductivity in the operating temperatures of an intermediate-temperature solid oxide fuel cell (IT-SOFC), relies on the presence of highly charged Re^6+^ cations distributed at random over the Co octahedral positions, preventing the highly repulsive conformations derived from the octahedral face-sharing involved in the hexagonal 2H polytypes [23]. The introduction of Re^6+^ cations also drives an electron-doping effect, enhancing a mixed valence over the Co cations and promoting the electrical conductivity. These structural features account for the successful use of SrCo_1−x_Re_x_O_3−*δ*_ as cathode materials in single test cells with pure H_2_ as a fuel, anticipated by the low electrode polarization resistance obtained by impedance spectroscopy in air, giving values as good as 0.080 Ω·cm^2^ and 0.066 Ω·cm^2^ for x = 0.05 and 0.1, respectively, at 850 °C.

## 4. Conclusions

We have studied the effect of the Re doping in the SrCoO_3−*δ*_ system, showing that SrCo_1−x_Re_x_O_3−*δ*_ (x = 0.05 and 0.10) oxides can be successfully utilized as cathode materials in single SOFC cells with LSGM as electrolyte. A maximum power density of 0.57 and 0.66 m·W·cm^−2^ was obtained at 850 °C with pure H_2_ as fuel for x = 0.05 and x = 0.10, respectively. The reasonable performance observed in the single cell tests can be correlated with the structural features obtained from NPD data collected in the usual working conditions of these cathodes in a SOFC. The crystal structure SrCo_0.9_Re_0.1_O_3−*δ*_ has been refined at RT in the tetragonal P4/mmm space group, and from 400 to 950 °C in the cubic Pm-3m space group. The presence of a large number of oxygen vacancies, *δ* = 0.351 at 850 °C, with high displacement factors, suggests high oxygen-ion mobility. The electrical conductivity in both samples (close to 50 S·cm^−1^ at 850 °C) seems to be enough to deliver a good performance. The polarization resistances in the electrode-electrolyte interface are small in both compounds, with ASR values as low as 0.065 Ω·cm^2^ and 0.087 Ω·cm^2^ for x = 0.10 and x = 0.05, respectively, at 850 °C. Although the thermal expansion is substantially higher than those usually displayed by SOFC electrolytes, there are no abrupt changes in the unit-cell volume, which increases smoothly over the entire temperature interval up to 900 °C. These characteristics make SrCo_1−x_Re_x_O_3−*δ*_ (x = 0.10 and 0.05) perovskites good candidate as cathode materials in intermediate-temperature SOFC.

## Figures and Tables

**Table 1:** Unit-cell, structural parameters and reliability factors for SrCo_0.90_Re_0.10_O_3−*δ*_ after the Rietveld refinement in the tetragonal P4/mmm space group, Z = 2, from neutron powder diffraction (NPD) data at room temperature (RT). a = 3.8676(1) Å, c = 7.7296(4) Å, V = 115.624(8) Å^3^.

Atoms	Site	x	y	z	B_iso_ (Å^2^)	B_eq_ (Å^2^) *	f_occ_
**Sr**	2*h*	1/2	1/2	0.2513(9)	1.14(2)		1.00
**Co1**	1*a*	0	0	0	0.09(4)		1.00
**(Co,Re)2**	1*b*	0	0	1/2	1.23(4)		0.815(1)/0.185(1)
**O1**	2*e*	1/2	0	0		1.139	0.921(2)
**O_2_**	2*g*	0	0	0.755(1)		2.227	1.00(2)
**O_3_**	2*f*	1/2	0	1/2		1.569	0.924(2)
* Anisotropic Thermal Factors (× 10^−4^) (*β*_12_ = *β*_13_ = *β*_23_ = 0)							
**Atoms**	*β*_11_	*β*_22_	*β*_33_				
**O1**	100(56)	151(59)	80(18)				
**O2**	401(30)	β_11_	79(13)				
**O3**	141(60)	207(67)	109(20)				

Reliability factors: *χ*^2^ = 2.41, R_p_(%) = 1.97, R_wp_(%) = 2.67, R_exp_(%) = 1.70, R_Bragg_(%) = 1.71.

**Table 2:** Unit-cell parameters, displacement parameters and reliability factors for SrCo_0.90_Re_0.10_O_3−*δ*_ in the cubic Pm-3m space group, refined from NPD data at 400, 600, 800 and 950 °C.

T (°C)	400	600	800	950
a (Å)	3.8979(4)	3.9221(1)	3.9460(1)	3.9641(1)
V (Å^3^)	59.227(1)	60.333(1)	61.442(2)	62.292(2)
**Atoms**				
**Sr**	**1*b* (½,½,½)**			
B_iso_ (Å^2^)	2.06(5)	2.59(6)	3.19(8)	3.78(9)
f_occ_	1.00	1.00	1.00	1.00
**(Co,Re)**	**1*a* (0,0,0)**			
B_iso_ (Å^2^)	0.50(8)	1.58(10)	2.12(13)	2.52(16)
f_occ_	0.9/0.1	0.9/0.1	0.9/0.1	0.9/0.1
**O**	**3*d* (½, 0, 0)**			
*β*_11_ *	317(13)	407(15)	534(19)	655(22)
*β*_22_ = *β*_33_ *	438(8)	638(10)	801(12)	943(14)
B*_eq_* (Å^2^)	2.415	3.461	4.456	5.324
f*_occ_*	2.733(2)	2.708(3)	2.649(34)	2.566(36)
**Reliability factors**				
*χ*^2^	2.49	1.56	1.40	1.37
*R_p_* (%)	1.60	1.40	1.33	1.31
*R_wp_* (%)	2.16	1.79	1.67	1.65
*R_Bragg_* (%)	1.65	2.52	2.11	2.14
**Bond distances (Å)**				
(Co,Re)–O (x6)	1.94899(2)	1.96105(2)	1.97299(4)	1.98205(5)

* Anisotropic thermal factors (×10^4^). *β*_12_ = *β*_13_ = *β*_23_ = 0.

**Table 3:** Area Specific Resistance (ASR) parameters obtained from the data fitting to the equivalent circuit shown in Figure 7 at different temperatures.

	700 °C	800 °C	850 °C
**SrCoO_3−*δ*_**			
R1/ASR (Ω·cm^2^)	4.065	0.592	0.326
**SrCo_0.95_Re_0.0_5O_3−*δ*_**			
R1 (Ω·cm^2^)	0.072	0.018	0.047
R2 (Ω·cm^2^)	0.585	0.102	0.033
ASR (Ω·cm^2^)	0.658	0.120	0.080
**SrCo_0.9_Re_0.1_O_3−*δ*_**			
R1 (Ω·cm^2^)	0.035	0.127	0.066
R2 (Ω·cm^2^)	0.565		
ASR (Ω·cm^2^)	0.600	0.127	0.066
